# Clinical outcomes of presbyopia-correcting intraocular lenses in patients with Fuchs endothelial corneal dystrophy

**DOI:** 10.1038/s41598-023-27830-x

**Published:** 2023-01-16

**Authors:** Michal Blau-Most, Olga Reitblat, Adi Levy, Ehud I. Assia, Guy Kleinmann

**Affiliations:** 1 Ein-Tal Eye Center, Tel Aviv, Israel; 2 Department of Ophthalmology, Meir Medical Center, 59 Tchernichovsky St., 44410 Kfar Sava, Israel; 3 Sackler Faculty of Medicine, Tel Aviv University, Tel Aviv, Israel; 4 Department of Ophthalmology, E. Wolfson Medical Center, Holon, Israel

**Keywords:** Outcomes research, Corneal diseases, Vision disorders

## Abstract

Fuchs endothelial corneal dystrophy (FECD) is considered a contraindication for the implantation of presbyopia-correcting IOLs, without sufficient corroborating evidence. A Retrospective, case–control study. Nineteen eyes of ten patients with grade 2–5 FECD (study group) and 57 healthy eyes of 57 patients (control group) who underwent cataract surgery with implantation of presbyopia-correcting IOLs, at the Ein-Tal Eye Center, Tel Aviv, Israel, were included. The target refraction was emmetropia for both groups. Two subgroups of IOLs were analyzed separately: extended depth of focus (EDOF), (9 eyes of FECD patients and 27 eyes of control patients) and multifocal IOLs (10 eyes of FECD patients and 30 eyes of control patients). Main outcome measures were visual acuity and refraction 6 weeks after the surgery. Secondary outcomes were patient perceptions of visual acuity, spectacle independence, photic phenomena and satisfaction scores, reported in a self-assessment questionnaire. FECD patients in the EDOF IOL subgroup had inferior uncorrected distance visual acuity (*P* = 0.007) and better uncorrected near visual acuity (*P* = 0.001) compared to the controls. They had less spectacle independence for the intermediate range (*P* = 0.01) and overall (*P* = 0.006). However, they did not have more photic phenomena. In the multifocal IOL subgroup, no significant differences were found between the FECD and the control group in visual acuity for all ranges and in spectacle independence. FECD patients had more photic phenomena than the controls (*P* = 0.006), but it did not interfere with daily life activities. There was no difference in post-operative mean spherical equivalent, patient reported visual perception, and general satisfaction between FECD and control patients in both groups. Our results suggest that presbyopia-correcting IOLs can be carefully considered in patients with grade 2–5 FECD, with slightly inferior results compared with healthy eyes.

## Introduction

Fuchs endothelial corneal dystrophy (FECD) is a common corneal dystrophy, characterized by endothelial cell loss and small excrescences of Descemet’s membrane (guttae), as early signs. The remaining endothelial cells stretch to fill the gaps leading to polymegethism and pleomorphism of the endothelial cells. In later stages, reduced activity of ion pumps and leaky barrier between the aqueous humor and the corneal stroma causes influx of water into the stroma. Corneal edema and reduced corneal sensation develop^1–3^.

The visual symptoms of FECD include halos and glare, caused by light scatter from the guttae and later, from the corneal edema. Decreased visual acuity with diurnal variation due to prominent corneal edema in the morning and reduced corneal edema during the day. This is followed by permanently decreased visual acuity^3,4^.

The immediate result of cataract surgery with monofocal intraocular lens (IOL) implantation is presbyopia. Several solutions were suggested for the induced presbyopia, such as mono-vision and presbyopia-correcting IOLs. Presbyopia-correcting IOLs improve the range of uncorrected visual acuity and reduce spectacle dependence. However, presbyopia-correcting IOLs may cause visual symptoms, such as reduced contrast sensitivity, halos, glare, and shadow and waxy vision^5–7^. The visual symptoms of presbyopia-correcting IOLs may exacerbate the similar visual symptoms of FECD. Therefore, they are considered as relative or absolute contraindications in FECD patients^5^. Moreover, cataract surgery in the presence of FECD is more challenging and there is increased risk of future need for corneal transplant.

In this study, we investigated the results and satisfaction of FECD patients who were implanted with presbyopia-correcting IOLs during routine cataract surgery.

## Materials and methods

This was a retrospective, case control study of FECD patients implanted with presbyopia-correcting IOLs (extended depth of focus (EDOF) or multifocal IOLs) during routine cataract surgery. The surgeries were performed by two experienced surgeons (GK and EIA), in the Ein-Tal Eye Center, Tel Aviv, Israel, between March 2012 and August 2018 (study group). The results were compared to those of an age- and IOL-matched control group, in a ratio of 1:3 (control group). A total of 57 healthy eyes of 57 patients who were operated during the same period by the same surgeons and were implanted with the same presbyopia-correcting IOLs, served as the control group.

The study adhered to the tenets of the Declaration of Helsinki, and was approved by the Meir Medical Center Institutional Ethics Committee. All patients undergoing cataract surgery with implantation of presbyopia correcting IOLs completed the questionnaire, regardless of this retrospective study, and therefore the Meir Medical Center Institutional Ethics Committee which approved the study waived the need to obtain informed consent.

FECD grading was based on the area and confluence of the guttae, and the presence of corneal edema, found during slit lamp bio-microscopy, in accordance with the grading described by Louttit et al. ^8^ (Table 1). FECD was considered mild when up to 12 central or paracentral nonconfluent guttae were noticed (grade 1), moderate when more than 12 nonconfluent guttae or confluent guttae up to an area of 5 mm (grade 2–5) were found and advanced when 5 mm or more of confluent guttae with corneal edema were present (grade 6).

**Table 1:** FECD Clinical Grading score (Louttit et al.^8^).

Grade	Central or paracentral guttae
1	≤ 12 scattered, nonconfluent
2	> 12 scattered, nonconfluent
3	1–2 mm (widest diameter), confluent
4	2–5 mm (widest diameter), confluent
5	> 5 mm (widest diameter), confluent
6	> 5 mm, confluent and with stromal or epithelial edema

The inclusion criteria for the study group were patients with moderate FECD without corneal edema (grade 2–5), implanted with presbyopia-correcting IOLs, uneventful cataract surgery and a follow-up visit at least six weeks after the surgery.

The exclusion criteria were mild FECD (grade 1) or advanced FECD with corneal edema (grade 6), other significant ocular morbidity and previous ocular surgeries. Eyes with significant irregular astigmatism were also excluded from presbyopia-correcting IOL implantation and from the study. The inclusion and exclusion criteria for the control group were the same, except for the presence of FECD.

The IOL power was calculated using biometric data (Lenstar LS900, Haag-Streit, Koeniz, Switzerland), third and fourth generation formulas (SRK/T, Holladay 1, Hoffer Q, Haigis, Olsen, and Barrett Universal II), and the Barrett online calculator for toric IOLs. We mainly used the Barrett result. The other formulas were used for confirmation and in cases with variability of the results between the different formulas. The target refraction was emmetropia or the nearest myopic alternative for both the FECD and the control patients. Corneal endothelial cell count was performed using an EM‐3000 specular microscope (Tomey Corporation, Nagoya, Japan).

The cataract surgeries were performed through a 2.2 to 2.4 mm clear corneal incision using phacoemulsification. Toric IOLs were considered for regular corneal astigmatism.

A questionnaire survey regarding perception of visual acuity, spectacle independence, photic phenomena and general satisfaction was completed at least one month after surgery. The answers were graded on a scale from 1 to 5, where 5 was an excellent result and 1 was a poor result.

The main outcome measurements were visual acuity and post-operative refraction at least six weeks after surgery. The secondary outcomes were patients’ perception of visual acuity and satisfaction scores, as reported in the self-assessment questionnaire.

### Statistical analysis

Data were analyzed using the SPSS software (version 21.0; IBM Corp., Armonk, NY). The Mann–Whitney U test was used to compare continuous variables between the two groups. Centroid astigmatism was analyzed by its double angled X and Y axis components. Paired Hotelling’s T-squared test was used for bivariate statistical analysis, as described by Naeser^9^ and indicated by Abulafia et al.^10^ Pearson’s Chi-Square or Fisher’s exact test, as indicated, were used for categorical variables. ANOVA test was used for multivariate regression analysis. Patient feedback from the survey was expressed as proportions and assessed as categorical variables. For analysis, positive outcomes regarding spectacle use were attributed to the answers "Never" and "Rarely". For questions regarding quality of vision, the answers "Good" and "Excellent" were considered indicative of high patient satisfaction. Photic phenomena were attributed to answers graded "Often" or "All the time" and overall patient satisfaction was graded as positive if the patient would choose or probably choose the same IOL again. A *P*-value of less than 0.05 was considered statistically significant, and a post hoc power analysis was conducted with G*Power 3.1.9.4 (Heinrich Heine University, Dusseldorf, Germany).

### Conference presentation

Presented at the European Society of Cataract & Refractive Surgeons Annual Meeting, September 16, 2019, Paris, France, and at the American Society of Cataract & Refractive Surgeons Virtual Annual Meeting, May 16, 2020.

## Results

A total of 19 eyes of 10 patients with FECD were included in the study (each eye was considered separately). The statistical power based on post hoc power analysis was 0.6. The mean endothelial cell count was 1,323.52 ± 767.54 cells/mm^2^ (range 0–2292 cells/mm^2)^, and the mean corneal thickness was 554.12 ± 22.77 microns (range 524–616 microns) in FECD patients. No correlation was found using a multivariate correlation analysis between endothelial cell count to corneal thickness and post operative visual acuity results for all ranges (distance, intermediate and near), (R^2^ = 0.65, *P* = 0.18). The aged-matched control group consisted of 57 healthy eyes of 57 patients who had cataract surgery and implantation of similar presbyopia-correcting IOLs (n = 57).

We analyzed two subgroups of IOLs separately: EDOF IOLs (9 eyes of FECD patients and 27 eyes of the control patients) and multifocal IOLs (10 eyes of FECD patients and 30 eyes of the control patients).

The demographic and ocular characteristics of the FECD patients and the control group before surgery were similar for the overall group as well as for the subgroups, except for shallower anterior chamber depth among the FECD patients in the multifocal IOL subgroup (3.18 ± 0.27 vs. 3.44 ± 0.34, respectively; *P* = 0.04). This can be explained by the trend toward older age in that subgroup, as the lens volume increases with age (68.8 ± 10.26 vs.62.4 ± 9.25; *P* = 0.054; Table 2).

**Table 2:** Baseline characteristics of the EDOF and multifocal IOLs subgroups.

Characteristic	EDOF IOLs (n = 36)			Multifocal IOLs (n = 40)			
	FECD (n = 9)	Control (n = 27)	*P* value	FECD (n = 10)		Control (n = 30)	*P* value
Sex M, n (%)	7 (77.8)	14 (51.9)	0.32	4 (40.0)		15 (50.0)	0.75
Age (y) mean ± SD	68.9 ± 8.4	65.1 ± 10.1	0.83	68.8 ± 10.26		62.4 ± 9.25	0.054
Eye RE, n (%)	5 (55.6)	12 (44.4)	0.74	5 (50.0)		16 (53.3)	0.75
Axial length (mm), mean ± SD	24.08 ± 0.82	24.15 ± 0.91	0.78	24.74 ± 1.12		24.41 ± 1.47	0.30
Anterior chamber depth (mm), mean ± SD	3.17 ± 0.21	3.24 ± 0.37	0.74	3.18 ± 0.27		3.44 ± 0.34	0.04
Average Keratometry (D), mean ± SD	43.3 ± 1.83	43.3 ± 1.27	0.90	43.16 ± 0.85		43.94 ± 1.31	0.17
IOL power (D), mean ± SD	20.28 ± 1.15	20.19 ± 3.09	0.56	17.95 ± 4.11		18.25 ± 3.85	0.80

*FECD* Fuchs endothelial corneal dystrophy; *IOLs* intraocular lenses; *M* male; *n* number; *y* years; *D* diopters.

Values are presented in mean ± SD, unless specified otherwise.

The multifocal subgroup was implanted with either FineVision trifocal IOL (PhysIOL Inc., Liège, Belgium) or AcrySof ReSTOR IOL (Alcon Laboratories, Inc., Fort Worth, TX). The EDOF subgroup included only the Tecnis Symfony IOL (Johnson and Johnson, New Brunswick, NJ); Table 3).

**Table 3:** Implanted IOLs.

Type of IOL	FECD group	Control group
FineVision trifocal IOL (n)	**4**	**12**
*Micro F (n)*	*2*	*7*
*POD FT (n)*	*2*	*5*
AcrySof ReSTOR (n)	**6**	**18**
*SN6AD1 (n)*	*5*	*18*
*SND1T4 (n)*	*1*	*0*
Tecnis Symfony IOL (n)	**9**	**27**
*ZXR00 (n)*	*3*	*12*
*ZXT (n)*	*6*	*15*

The visual acuity and post-operative refraction data were taken from the follow-up visit 6 weeks after the surgery.

## Visual acuity results

### EDOF IOLs

The mean post-operative spherical equivalent (SE) of the FECD patients implanted with EDOF IOLs and of the healthy control patients was similar: − 0.26 ± 0.33 and − 0.24 ± 0.24 (D), respectively (*P* = 0.81). The mean SE error (The aimed SE- the actual SE) was similar between the FECD patients and the control patients (0.08 ± 0.31 and 0.03 ± 0.26; *P* = 0.30, respectively).

The average postoperative sphere, astigmatism and axis of FECD patients versus the control patients were also similar (0.0 ± 0.33 vs. − 0.03 ± 0.24; *P* = 0.79; − 0.53 ± 0.4 vs. − 0.42 ± 0.35; *P* = 0.43; 100.1 ± 50 vs. 70 ± 64; *P* = 0.20; respectively).

**Figure 1:**
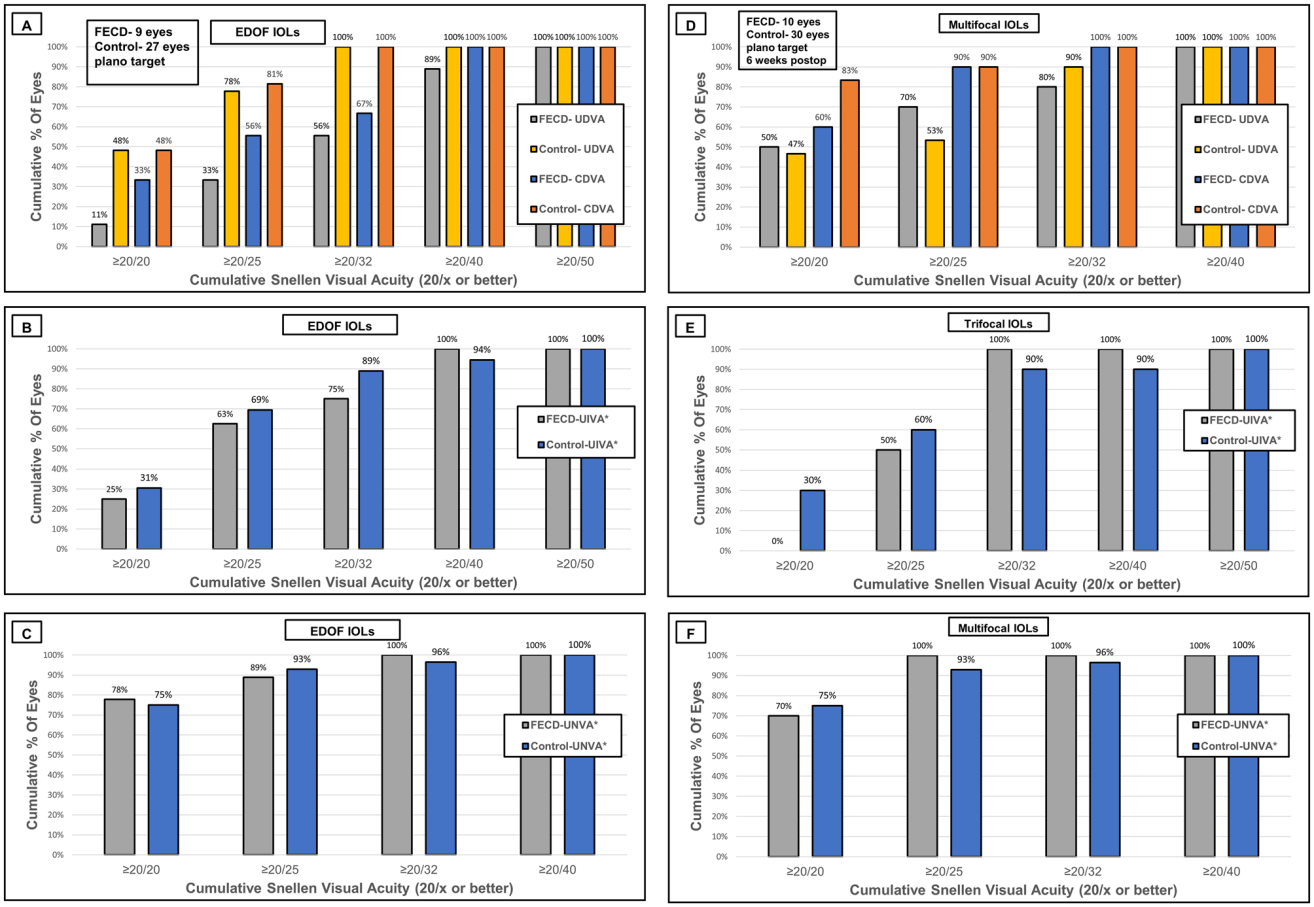
Refractive outcomes *EDOF IOL subgroup*. (**A**) UDVA and CDVA (Snellen), (**B**) UIVA (Snellen), measured at 80 cm, (**C**) UNVA (Snellen), measured at 40 cm *Multifocal IOL subgroup,* (**D**) UDVA and CDVA (Snellen), (**E**) UIVA (Snellen), measured at 80 cm, (**F**) UNVA (Snellen) measured at 40 cm (EDOF = extended depth of focus, IOLs = intraocular lenses, UDVA = uncorrected distance visual acuity, CDVA = corrected distance visual acuity, UIVA = uncorrected intermediate visual acuity, UNVA = uncorrected near visual acuity, FECD = Fuchs endothelial corneal dystrophy).

The mean uncorrected distance visual acuity (UDVA) (LogMAR) of the FECD patients was inferior compared with the healthy control patients (mean UDVA was 0.17 ± 0.04 (Snellen 20/30) and 0.04 ± 0.02 (Snellen 20/22), respectively; *P* = 0.007). UDVA of 20/40 (Snellen) or better was found in 89% of the FECD eyes and in 100% of the healthy control eyes (Table 4; Fig. 1A). There was no difference in uncorrected intermediate visual acuity (UIVA) between FECD and the control group (*P* = 0.50).

**Table 4:** Visual acuity results.

	UDVA	CDVA	UIVA	UNVA
EDOF IOLs subgroup				
**FECD**	0.17 ± 0.04 (20/30)	0.12 ± 0.04 (20/26)	0.14 ± 0.04 (20/28)	0.04 ± 0.02 (20/22)
Control	0.04 ± 0.02 (20/22)	0.03 ± 0.01 (20/21)	0.1 ± 0.02 (20/25)	0.26 ± 0.04 (20/36)
*P*-value	0.007	0.06	0.50	0.001
Multifocal IOLs subgroup				
**FECD**	0.06 ± 0.11 (20/23)	0.03 ± 0.08 (20/21)	0.11 ± 0.14 (20/26)	0.03 ± 0.07 (20/21)
Control	0.08 ± 0.10 (20/24)	0.02 ± 0.08 (20/21)	0.11 ± 0.10 (20/26)	0.03 ± 0.05 (20/21)
*P*-value	0.58	0.91	0.83	0.88

The visual acuity results are displayed as mean ± SD in LogMAR and Snellen in parentheses.

*IOLs* intraocular lenses; *EDOF* extended depth of focus; *FECD* Fuchs endothelial corneal dystrophy; *UDVA* uncorrected distance visual acuity; *CDVA* corrected distance visual acuity; *UIVA* uncorrected intermediate visual acuity; *UNVA* uncorrected near visual acuity. UIVA for the multifocal IOLs subgroup consisted of trifocal IOLs only.

Uncorrected near visual acuity (UNVA) among the FECD patients implanted with EDOF IOLs was better than that of the healthy control eyes (mean 0.04 ± 0.02 (Snellen 20/22) and 0.26 ± 0.04 (Snellen 20/36), respectively, *P* = 0.001).

### Multifocal IOLs

The mean post-operative SE of the FECD eyes implanted with multifocal IOLs and of the healthy control eyes was similar (− 0.03 ± 0.26 and − 0.18 ± 0.25, respectively; *P* = 0.12). The mean SE error (The aimed SE- the actual SE) was similar between the FECD patients and the control patients (0.13 ± 0.22 and 0.03 ± 0.28; *P* = 0.12, respectively.) Additionally, the average postoperative sphere, astigmatism and axis of the FECD patients versus the control patients were also similar (0.2 ± 0.31 vs. 0.03 ± 0.28; *P* = 0.13; − 0.45 ± 0.28 vs. − 0.43 ± 0.34; *P* = 0.89; 104.5 ± 62 vs. 92.8 ± 50; *P* = 0.55; respectively).

There was no significant difference in visual acuity between the FECD eyes the matched healthy control eyes for all distances (Table 4, Fig. 1).

**Figure 2:**
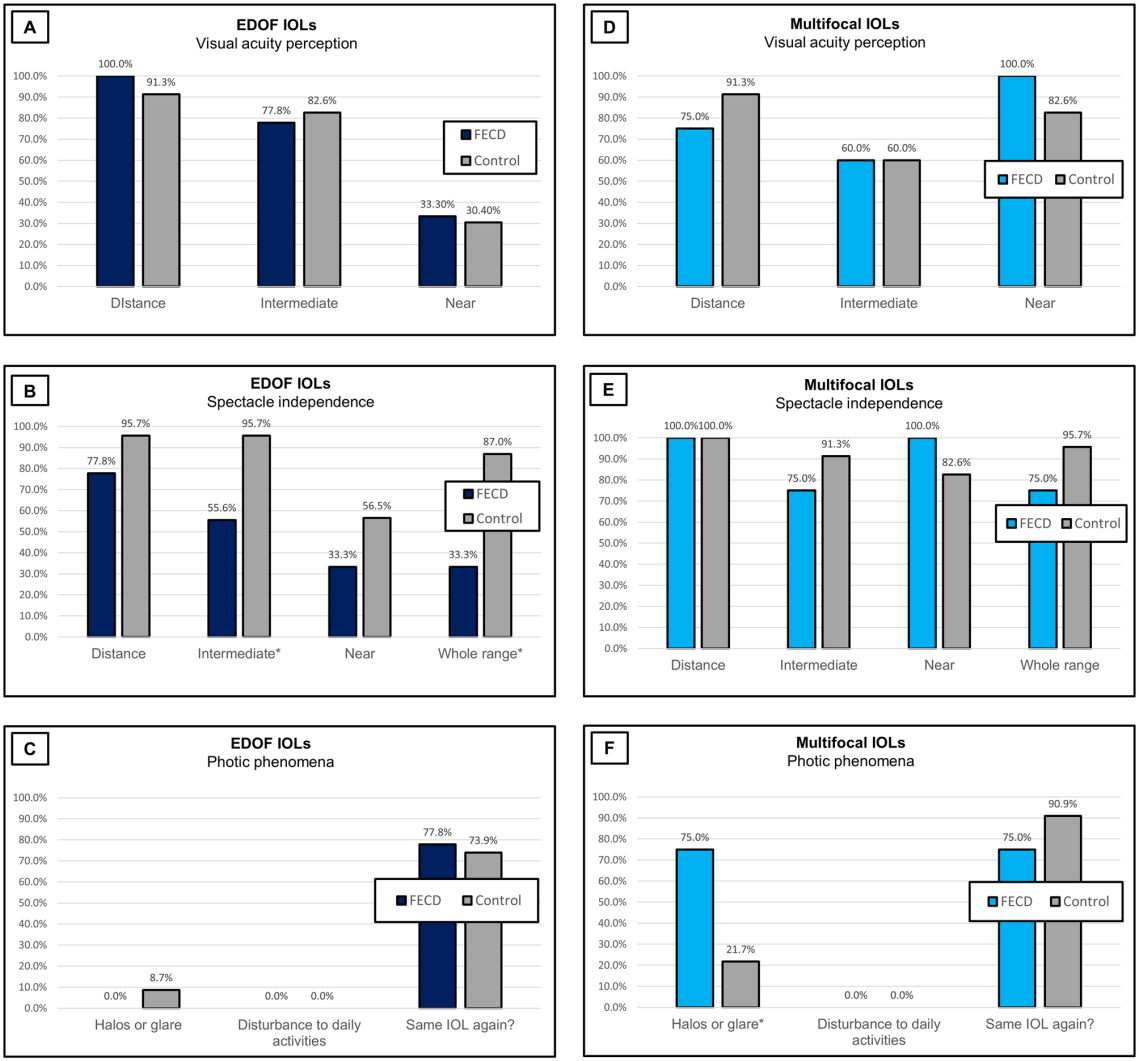
Patients’ self-assessment questionnaire results (**A**, **D).** Visual acuity perception. The percentage of patients in each group who rated their uncorrected visual acuity as “excellent” or “good” for distance, intermediate and near. (**A** EDOF IOL subgroup, **D** Multifocal IOLs subgroup). (**B**, **E).** Spectacle independence. The percentage of patients in each group who reported they “never” or “rarely” use spectacles for distance, intermediate, near and overall. (**B** EDOF IOLs subgroup, **E**- Multifocal IOLs subgroup). (**C**, **F)** Photic phenomena. The percentage of patients in each group who reported having halos or glare, and the percentage of patients in each group who reported disturbances in daily life activity from the halos or glare “often” or “all the time”. Satisfaction—the percentage of patients in each group who answered “probably” or “yes” to the question: “Would you choose the same IOL again?”. (**C**. EDOF IOLs subgroup, **F**. Multifocal IOLs subgroup).*Statistically significant (IOLs = intraocular lenses, EDOF = extended depth of focus, FECD = Fuchs endothelial corneal dystrophy).

## Astigmatism

The FECD patients in the EDOF-toric IOL subgroup, had higher preoperative and postoperative astigmatism compared with the control group (*P* < 0.001, *P* = 0.03, respectively, Table 5).

**Table 5:** Preoperative and postoperative astigmatism in patients with **PC-toric-IOLs**.

Variable	FECD	Control	*P* value
*EDOF IOLs subgroup*			
Pre-op astigmatism (D), mean ± SD			
Magnitude	3.45 ± 1.43	1.53 ± 0.74	
Centroid	2.51 ± 3.03@95°	0.7 ± 1.6@14°	< 0.001
Post-op astigmatism (D), mean ± SD			
Magnitude	0.54 ± 0.44	0.32 ± 0.3	
Centroid	0.42 ± 0.6@30°	0.19 ± 0.4@105°	0.03
*Multifocal IOL subgroup*			
Pre-op astigmatism (D), mean ± SD			
Magnitude	1.1 ± 0.33	1.46 ± 0.79	
Centroid	0.56 ± 1.23@30°	0.70 ± 1.68@76°	0.73
Post-op astigmatism (D), mean ± SD			
Magnitude	0.59 ± 0.32	0.20 ± 0.10	
Centroid	0.53 ± 0.50@5°	0.02 ± 0.25@151°	0.13

*D* diopters.

When calculating the astigmatism for the whole EDOF IOL subgroup (toric and non toric IOLs), the FECD patients had a trend toward higher preoperative corneal astigmatism magnitude (2.52 ± 1.86 and 1.18 ± 0.75, respectively; *P* = 0.08), and were implanted with a higher number of toric IOLs compared to the control (Table 3). There was no difference in the postoperative residual refractive astigmatism magnitude between the FECD patients and the healthy control group implanted with EDOF IOLs (0.53 ± 0.4 and 0.42 ± 0.35, respectively; *P* = 0.52).

In the multifocal-toric IOL subgroup, there was no difference in the preoperative and postoperative astigmatism between the FECD and the control groups (*P* = 0.73 and *P* = 0.13, respectively, Table 5).

When calculating the astigmatism for the whole multifocal IOL subgroup (toric and non toric IOLs), there was a higher preoperative corneal astigmatism magnitude in FECD patients (1.13 ± 0.27 and 0.72 ± 0.57, respectively; *P* = 0.002), and they were implanted with a higher number of toric IOLs compared to the control group (Table 3).There was no difference in the postoperative residual refractive astigmatism magnitude between FECD and the control patients implanted with multifocal IOLs (0.45 ± 0.28 and 0.43 ± 0.34, respectively; *P* = 0.61).

## Questionnaire

### EDOF IOLs

Nine FECD patients (100%) and 15 control patients (85%) completed the post-operative questionnaire. There was no difference in visual acuity perception between the FECD patients and the control group for all the ranges (distance, *P* = 0.36; intermediate, *P* = 0.75; near, *P* = 0.87; Fig. 2A), as well as no difference in spectacle independence for the distance (*P* = 0.18) and near ranges(*P* = 0.43) (Fig. 2B). The control patients reported significantly better spectacle independence for the intermediate range (*P* = 0.01) and overall (*P* = 0.006) (Fig. 2B), There was no difference in the photopic phenomena between the FECD and the healthy control group (*P* = 0.36; Fig. 2C). General satisfaction, represented as the desire to implant the same IOL again, was also similar between those groups (*P* = 0.82; Fig. 2C).

### Multifocal IOLs

Eight FECD patients (80%) and 23 control patients (77%), completed the post-operative questionnaire. There was no difference in visual acuity perception (distance, *P* = 0.24; intermediate, *P* = 0.96; near, *P* = 0.20; Fig. 2D), or spectacle independence (distance, P > 0.99; intermediate, *P* = 0.27; near, *P* = 0.55; whole range, *P* = 0.16; Fig. 2E) between the FECD patients and the healthy control patients. Seventy-five percent of the FECD patients implanted with multifocal IOLs reported halos and glare often or all the time, as compared to 21.7% in their matched control group (*P* = 0.006; Fig. 2F). However, it did not disturb their daily life activities, and the general satisfaction was similar between the groups (*P* = 0.16 and *P* = 0.28, respectively; Fig. 2F).

The mean follow-up time for the FECD group was 2.8 ± 1.8 years (range 1.25–7 years). None of the patients required corneal transplantation or IOL exchange during the follow-up period.

## Discussion

In this study, we found that implantation of presbyopia-correcting IOLs (EDOF and multifocal IOLs), during routine cataract surgery in patients with grade 2 to 5 FECD, who are not candidates for corneal transplant, can be carefully considered. The results were slightly inferior compared with healthy age-matched eyes.

Viberg et al.^11^ performed a large, population-based study of 33,741 patients (based on data from the Swedish National Cataract Registry), and included 893 patients with guttae who underwent cataract surgery. Both FECD and control patients had improved corrected distance visual acuity and self-assessed visual outcomes. However, the patients with guttae had inferior objective visual acuity, as well as inferior self-assessed visual outcomes in comparison with the control group with healthy eyes (*P* < 0.001). Watanabe et al.^12^ found that the area of the corneal guttae was correlated to the corrected distance visual acuity, contrast sensitivity, and intraocular stray light among patients with mild FECD without corneal edema. They concluded that corneal guttae caused poorer quality of vision in eyes with mild FECD.

FECD patients demonstrated a trend toward higher preoperative corneal astigmatism magnitude in the EDOF subgroup (*P* = 0.08) and higher preoperative corneal astigmatism magnitude in the multifocal group (*P* = 0.002). However, with implantation of toric IOLs, the postoperative residual refractive astigmatism magnitude in both the EDOF IOLs and the multifocal IOLs groups were similar for the FECD and the control patients (*P* = 0.52, *P* = 0.61, respectively). The only subgroup with higher residual refractive astigmatism was the EDOF-toric-IOL subgroup, in which the postoperative refractive astigmatism among FECD patients was significantly higher compared with the healthy controls (*P* < 0.001 vs. *P* = 0.03, respectively; Table 5). It may explain the inferior UDVA (*P* = 0.007), and the better UNVA (*P* = 0.001) among the FECD patients implanted with EDOF IOLs, and be attributed to the EDOF-toric-IOLs subgroup that had higher postoperative residual astigmatism, and hence a mild myopic refraction.

However, these differences were probably not clinically significant, as reflected by similar reported visual perception for all ranges between the groups. In the EDOF IOLs subgroup, the FECD patients reported less spectacle independence for intermediate range (*P* = 0.01) and overall (*P* = 0.006), although the objective visual acuity results were similar (*P* = 0.50). This may be related to the limitations of the objective visual acuity examinations in demonstrating parameters such as contrast sensitivity and color contrast. The FECD patients implanted with EDOF IOLs did not have more photopic phenomena compared to the control group, and none of them reported halos and glare “often” or “all the time” (*P* = 0.36, Fig. 2C).

Results reported in the literature are inconsistent regarding halos and glare in EDOF IOLs compared to monofocal IOLs. A European, multicenter, prospective study found that 4 months after cataract surgery, 51 of 68 patients (75%) implanted with EDOF IOLs did not have halos and glare, using a halos and glare simulator^13^. Another study based on data from the US FDA premarket approval trials, found more halos and glare with multifocal and EDOF IOLs compared to monofocal IOLs^14^. Finally, in a meta-analysis by Liu et al.^15^, two studies found no significant difference in halos and glare between EDOF and monofocal IOLs, while another US FDA study of 295 eyes implanted with TECNIS Symfony EDOF and monofocal lenses, found more frequent halos and glare in EDOF compared to monofocal IOLs.

In the multifocal IOLs subgroup, there was no difference in the objective visual acuity, post-operative mean spherical equivalent, visual acuity perception and spectacle independence between the Fuchs and control patients for all ranges (Fig. 1D-F, Fig. 2D-E). However, the rate of positive dysphotopsia was significantly higher in the Fuchs patients compared to the healthy controls (*P* = 0.006, Fig. 2F). As many as 75% of the FECD patients implanted with multifocal IOLs experienced halos and glare “often” or “all the time”. However, none reported that it disturbed their daily life activity “often” or “all the time”, and there was no significant difference in the overall satisfaction between the groups (Fig. 2F).

It is well-described that multifocal IOLs can cause significant photic phenomena and reduced night vision, even among healthy patients^16–19^. Wilkins et al. ^20^ conducted a randomized trial comparing monovision to multifocal IOLs after bilateral cataract surgery in 187 eyes, and found that 43% of the healthy patients who were implanted with multifocal IOLs, reported “annoying” or “debilitating” dysphotopsia, as compared to 18% in the monovision group (*P* < 0.001). These findings are similar to our own experience in healthy eyes. Using the same questionnaire and setup, we found that more than a third of the healthy patients implanted with trifocal IOLs reported photic phenomena “often” or “all the time”^21^.

In our study, the percentage of photopic phenomena in the FECD patients implanted with multifocal IOLs is higher than that described in the literature for healthy eyes^20,21^. It is reasonable to assume that the combination of corneal pathology with the known halos and glare limitation of multifocal IOLs may exacerbate the photic phenomena in the FECD patients more than in the healthy eyes. Nevertheless, as mentioned, none of the patients reported that it interfered significantly with daily life activities, and no IOL was exchanged.

A recent study reported the results of presbyopia-correcting IOLs after descemet membrane endothelial keratoplasty (DMEK) in FECD patients^22^. In this study, the median time between DMEK and the cataract surgery was 5 months, and it was offered as an alternative to combined endothelial keratoplasty and cataract surgery, or as an alternative to cataract surgery before endothelial keratoplasty. The authors reported better postoperative refractive results when DMEK was performed before cataract surgery, because the biometry measurements and IOL calculations were more accurate after the corneal guttae and edema were removed.

Indeed, there is ongoing concern regarding the need for future corneal transplant in FECD patients due to the cataract surgery or due to the natural history and progress of the FECD that may jeopardize the effectiveness of the presbyopia-correcting IOL. Viberg et al.^23^ assessed the incidence rate of corneal transplantation after phacoemulsification based on data from 276,362 cataract patients from the Swedish National Cataract Registry. Among 3338 patients with corneal guttae, 152 (4.6%) underwent corneal transplantation, and among 188,915 patients without corneal guttae, 141 (0.1%), underwent corneal transplantation. Although the relative risk for corneal transplantation after phacoemulsification was 68.2 times higher in patients with corneal guttae, as compared to healthy controls, more than 95% of the patients with corneal guttae did not require corneal transplantation, during a 7-year follow-up. Moreover, progress in lamellar corneal transplant surgery led to minimal influence on the patients’ refractive status. These findings raise a discussion regarding implantation of presbyopia-correcting IOLs in FECD patients^24^. This discussion should be conducted carefully, with full disclosure of the inferiority of the results in comparison to the general healthy population and the increased incidence of photic phenomena.

To optimize the results and to avoid presbyopia-correcting IOLs in eyes that are candidates for keratoplasty, Van Cleynenbreugel et al.^25^ recommended evaluating the central corneal thickness and backscatter at the basal epithelial layer, preoperatively. They claim that it can help predict the need for future endothelial keratoplasty. In our study, none of the patients required corneal transplantation during a mean follow-up of more than two and a half years.

The limitations of the current study include its small sample size (the statistical power based on post hoc power analysis was only 0.6), and its retrospective nature. Due to the small sample size, IOLs with different optical characteristics (bifocal and tri-focal) were analyzed together in the multifocal subgroup. Furthermore, the preoperative and postoperative astigmatism was higher among FECD patients implanted with EDOF-toric-IOLs (Table 5), and more toric-IOLs were implanted in FECD patients, as compared to the control group. Additionally, we did not examine the CDIVA and the CDNVA, which could have eliminated the postoperative refractive error, especially in the EDOF-toric-IOLs group that had significantly higher residual astigmatism and more myopic refraction. We also included two eyes of each FECD patient when we could, as opposed to only one eye of each healthy control, in order to increase the sample size, and it could have affected the subjective visual acuity results by confounding between fellow eyes.

In conclusion, while the FECD patients implanted with EDOF IOLs had inferior UDVA and less spectacles independence, they did not have more photic phenomena compared with the healthy control eyes. The contrary was found in the multifocal IOLs subgroup with similar UDVA and spectacle independence results, but significantly more photic phenomena, in the FECD patients, compared with the healthy control eyes. This information can help tailor IOL selection for different individuals with different needs. However, caution should be taken with extrapolating our results to other presbyopia-correcting IOLs that were not investigated in this study. The FECD patients in both subgroups reported high general satisfaction, similarly to the control group, as indicated by their responses to the question of whether they would choose the same IOL again. Therefore, presbyopia-correcting IOLs can be carefully considered in patients with FECD, without corneal edema, who are not candidates for corneal transplantation, with the above-mentioned reservations. A large, randomized control study is warranted to support our findings.

## Supplementary Information

Supplementary Information.

## References

[1] Vedana G (2016). Fuchs endothelial corneal dystrophy : Current perspectives. Clin. Ophthalmol..

[2] Iovieno A, Neri A, Soldani AM, Adani C, Fontana L (2017). Descemetorhexis without graft placement for the treatment of fuchs endothelial dystrophy: Preliminary results and review of the literature. Cornea.

[3] Elhalis H, Azizi B, Jurkunas UV (2010). Fuchs endothelial corneal dystrophy. Ocul Surf..

[4] Wacker K, Baratz KH, Bourne WM, Patel SV (2018). Patient-reported visual disability in Fuchs ’ endothelial corneal dystrophy measured by the visual function and corneal health status instrument. Ophthalmology.

[5] Braga-Mele R, Chang D, Dewey S, Foster G, Henderson BA, Hill W (2014). Multifocal intraocular lenses: Relative indications and contraindications for implantation. J. Cataract Refract Surg..

[6] Rosen E, Alió JL, Dick HB, Dell S, Slade S (2016). Efficacy and safety of multifocal intraocular lenses following cataract and refractive lens exchange: Metaanalysis of peer-reviewed publications. J. Cataract Refract. Surg..

[7] Hovanesian JA (2018). Patient-reported outcomes of multifocal and accommodating intraocular lenses: Analysis of 117 patients 2–10 years after surgery. Clin Ophthalmol..

[8] Louttit MD, Kopplin LJ, Igo RPJ, Fondran JR, Tagliaferri A, Bardenstein D (2012). A multicenter study to map genes for Fuchs endothelial corneal dystrophy: Baseline characteristics and heritability. Cornea.

[9] Næser K (2008). Assessment and statistics of surgically induced astigmatism. Acta Ophthalmol..

[10] Abulafia A, Koch DD, Holladay JT, Wang L, Hill W (2018). Pursuing perfection in intraocular lens calculations IV. Rethinking astigmatism analysis for intraocular lens-based surgery: Suggested terminology, analysis, and standards for outcome reports. J. Cataract Refract. Surg..

[11] Viberg A, Liv P, Behndig A, Lundström M, Byström B (2019). The impact of corneal guttata on the results of cataract surgery. J. Cataract Refract. Surg..

[12] Watanabe S, Oie Y, Fujimoto H, Soma T (2015). Relationship between corneal Guttae and quality of vision in patients with Mild Fuchs ’ endothelial corneal dystrophy. Ophthalmology.

[13] Auffarth GU, Moraru O, Munteanu M, Tognetto D. Non-comparative clinical evaluation of an. 2020. 10.3928/1081597X-20200603-01 32644164

[14] Schallhorn JM (2021). Multifocal and extended depth of focus intraocular lenses: A comparison of data from the united states food and drug administration premarket approval trials. J. Refract Surg..

[15] Liu J, Dong Y, Wang Y (2019). Efficacy and safety of extended depth of focus intraocular lenses in cataract surgery: A systematic review and meta-analysis. BMC Ophthalmol..

[16] Woodward MA, Randleman JB, Stulting RD (2009). Dissatisfaction after multifocal intraocular lens implantation. J. Cataract Refract. Surg..

[17] Kretz FTA, Choi CY, Müller M, Gerl M, Gerl RH, Auffarth GU (2016). Visual outcomes, patient satisfaction and spectacle independence with a trifocal diffractive intraocular lens. Korean J. Ophthalmol..

[18] Bilbao-Calabuig R, Llovet-Rausell A, Ortega-Usobiaga J, Martínez-del-Pozo M, Mayordomo-Cerdá F, Segura-Albentosa C (2017). Visual outcomes following bilateral lmplantation of two diffractive trifocal intraocular lenses in 10 084 eyes. Am. J. Ophthalmol..

[19] Ganesh S, Brar S, Pawar A (2017). Long-term visual outcomes and patient satisfaction following bilateral implantation of trifocal intraocular lenses. Clin. Ophthalmol..

[20] Wilkins MR, Allan BD, Rubin GS, Findl O, Hollick EJ, Bunce C (2013). Randomized trial of multifocal intraocular lenses versus monovision after bilateral cataract surgery. Ophthalmology.

[21] Rodov L, Reitblat O, Levy A, Assia EI, Kleinmann G (2019). Visual outcomes and patient satisfaction for trifocal, extended depth of focus and monofocal intraocular lenses. J. Refract. Surg..

[22] Price MO, Pinkus D, Price FWJ (2020). Implantation of presbyopia-correcting intraocular lenses staged after descemet membrane endothelial Keratoplasty in patients with Fuchs dystrophy. Cornea.

[23] Viberg A, Samolov B, Claesson Armitage M, Behndig A, Byström B (2020). Incidence of corneal transplantation after phacoemulsification in patients with corneal guttata: A registry-based cohort study. J. Cataract Refract. Surg..

[24] Deng SX, Lee WB, Hammersmith KM, Kuo AN, Li JY, Shen JF (2018). Descemet membrane endothelial Keratoplasty: Safety and outcomes: A report by the American academy of ophthalmology. Ophthalmology.

[25] Van Cleynenbreugel H, Remeijer L, Hillenaar T (2014). Cataract surgery in patients with fuchs’ endothelial corneal dystrophy: When to consider a triple procedure. Ophthalmology.

