# Supplementary material for: Clinical outcomes of presbyopia-correcting intraocular lenses in patients with Fuchs endothelial corneal dystrophy
Source: Sci Rep. 2023 Jan 16;13:786. doi: 10.1038/s41598-023-27830-x (PMC9842705; doi:10.1038/s41598-023-27830-x)
Supplement: Supplementary file 1 — Supplementary Information. [file 41598_2023_27830_MOESM1_ESM.docx]

**Appendix****- patients’ self-assessment questionnaire**

|  | **FECD, n (%)** | **Healthy eyes, n (%)** | **P-value** |
| --- | --- | --- | --- |
| **Q1 - How would you rate your distance vision?** | | | 0.66 |
| Very poor | 0 (0.0) | 0 (0.0) |  |
| Poor | 0 (0.0) | 0 (0.0) |  |
| Fair | 2 (11.8) | 4 (8.7) |  |
| Good | 8 (47.1) | 13 (28.3) |  |
| Excellent | 7 (41.2) | 29 (63.0) |  |
| **Q2 - How would you rate your intermediate vision?** | | | 0.74 |
| *Very poor* | 0 (0.0) | 0 (0.0) |  |
| *Poor* | 2 (11.8) | 2 (11.8) |  |
| *Fair* | 2 (11.8) | 7 (15.2) |  |
| *Good* | 8 (47.1) | 14 (30.4) |  |
| *Excellent* | 5 (29.4) | 23 (50.0) |  |
| **Q3 - How would you rate your near vision?** | | | 0.56 |
| *Very poor* | 0 (0.0) | 4 (8.7) |  |
| *Poor* | 4 (23.5) | 7 (15.2) |  |
| *Fair* | 2 (11.8) | 9 (19.6) |  |
| *Good* | 4 (23.5) | 6 (13.0) |  |
| *Excellent* | 7 (41.2) | 20 (43.5) |  |
| **Q4 - How frequently do you use glasses for distance vision?** | | | 0.18 |
| *All the time* | 0 (0.0) | 1 (2.2) |  |
| *Often* | 2 (11.8) | 0 (0.0) |  |
| *Sometimes* | 0 (0.0) | 0 (0.0) |  |
| *Rarely* | 1 (5.9) | 3 (6.5) |  |
| *Never* | 14 (82.4) | 42 (91.3) |  |
| **Q5 - How frequently do you use glasses for intermediate vision?** | | | 0.009* |
| *All the time* | 0 (0.0) | 1 (2.2) |  |
| *Often* | 6 (35.3) | 0 (0.0) |  |
| *Sometimes* | 0 (0.0) | 2 (4.3) |  |
| *Rarely* | 0 (0.0) | 2 (4.3) |  |
| *Never* | 11 (64.7) | 41 (89.1) |  |
| **Q6 - How frequently do you use glasses for near distance vision?** | | | 0.71 |
| *All the time* | 4 (23.5) | 5 (10.9) |  |
| *Often* | 2 (11.8) | 5 (10.9) |  |
| *Sometimes* | 0 (0.0) | 4 (8.7) |  |
| *Rarely* | 1 (5.9) | 4 (8.7) |  |
| *Never* | 10 (58.8) | 28 (60.9) |  |
| **Q7 - How frequently do you use glasses for any distance?** | | | 0.002* |
| *All the time* | 2 (11.8) | 0 (0.0) |  |
| *Often* | 4 (23.5) | 1 (2.2) |  |
| *Sometimes* | 2 (11.8) | 3 (6.5) |  |
| *Rarely* | 1 (5.9) | 14 (30.4) |  |
| *Never* | 8 (47.1) | 28 (60.9) |  |
| **Q8 – Do you experience halos or glare in your vision?** | | | 0.03* |
| *All the time* | 0 (0.0) | 0 (0.0) |  |
| *Often* | 6 (35.3) | 2 (4.3) |  |
| *Sometimes* | 3 (6.5) | 9 (19.6) |  |
| *Rarely* | 2 (11.8) | 8 (17.4) |  |
| *Never* | 6 (35.3) | 27 (58.7) |  |
| **Q9 – Do the halos and glare disturb your daily activities?** | | | 0.30 |
| *All the time* | 0 (0.0) | 0 (0.0) |  |
| *Often* | 0 (0.0) | 0 (0.0) |  |
| *Sometimes* | 2 (11.8) | 2 (4.3) |  |
| *Rarely* | 7 (41.2) | 3 (6.5) |  |
| *Never* | 8 (47.1) | 41 (89.1) |  |
| **Q10 - Would you choose the same IOL again?** | | | 0.72 |
| *No* | 0 (0.0) | 1 (2.2) |  |
| *Probably not* | 0 (0.0) | 0 (0.0) |  |
| *I don’t know* | 4 (23.5) | 7 (15.6) |  |
| *Probably* | 6 (35.3) | 4 (8.9) |  |
| *Yes* | 7 (41.2) | 33 (73.3) |  |

*Statistically significant
